# Addressing Overlooked Variables in Preclinical Fracture Repair Research for Improved Translation

**DOI:** 10.1002/jor.70268

**Published:** 2026-08-26

**Authors:** Christina Capobianco, Katharina Schmidt‐Bleek, Melanie Haffner‐Luntzer, Chelsea Bahney, Joseph L. Roberts, Matthew J. Silva, Annemarie Lang

**Affiliations:** ^1^ Department of Orthopaedic Surgery University of Michigan Ann Arbor Michigan USA; ^2^ Department of Biomedical Engineering University of Michigan Ann Arbor Michigan USA; ^3^ Julius Wolff Institute – Center for Musculoskeletal Biomechanics and Regeneration BIH @ Charité – Universitätsmedizin Berlin Berlin Germany; ^4^ Institute of Orthopaedic Research and Biomechanics Ulm University Medical Center Ulm Germany; ^5^ Department of Orthopaedic Surgery University of California San Francisco San Francisco California USA; ^6^ College of Health Solutions Arizona State University Phoenix Arizona USA; ^7^ Biodesign Institute Center for Health Through Microbiomes Arizona State University Tempe Arizona USA; ^8^ Department of Orthopaedics Washington University School of Medicine St. Louis Missouri USA

**Keywords:** bone regeneration, experimental variables, fracture healing, preclinical animal models

## Abstract

In preclinical research focused on bone fracture repair, a significant gap exists in understanding and incorporating key variables that influence homeostasis and pathophysiological processes. Factors such as immune system responses, sex differences, pain and innervation, and microbiota are often overlooked. Preclinical studies frequently rely on rodent models housed under sterile, specific pathogen‐free conditions, resulting in naïve immune responses. Additionally, these studies typically use only one sex, fail to monitor stress behaviors, and do not assess infections or commensal microorganisms. While such controlled approaches enable the study of essential mechanisms, they may hinder the successful translation of findings to immunocompetent and diverse human populations, who do not live in sterile environments and vary widely in biological sex, gender, age, immune history, and living conditions. Here, we summarize the material presented at the Preclinical Models Section and International Section of Fracture Repair Workshop held during the 2026 Orthopaedic Research Society (ORS) Annual Meeting in Charlotte, North Carolina.

## 1 Introduction

Delayed and non‐union fracture repair remains an unresolved clinical problem. Of the 160–190 million new bone fractures that occur annually [1], it is commonly accepted that approximately 5%–10% undergo delayed or non‐union healing [2]. However, a recent multi‐center study suggests that this may be an underestimate, as it shows that the actual incidence of delayed healing is more than twice as high [3]. Assessment of fracture readmission of patients within 2 years of injury likewise revealed an 8.1% readmission rate with notably higher complications for femoral (13.6%) and tibial shaft (11.7%) fractures [4]. This highlights a critical need for clinically relevant preclinical models that capture environmental factors contributing to poor healing in patient populations.

Long bone fracture repair is a well‐established, complex process that begins with the inflammatory phase and involves several critical steps. The acute inflammatory phase, occurring within the first 5 days post‐fracture, is critical for initiating the regenerative repair process. During this time, inflammatory cells clear debris and recruit stromal progenitors, which deposit matrix and differentiate to form the soft fibrovascular/cartilaginous callus. The bony callus forms within 6 weeks post‐fracture in humans through two parallel processes: endochondral ossification, in which the cartilaginous template is mineralized, and intramembranous ossification, in which stromal progenitors differentiate directly into osteoblasts and deposit matrix. Over the following weeks and up to 1 year, the callus is gradually reorganized with mature mineralized tissue according to mechanical strains as the bone is restored to its original structure and shape [5, 6]. Failure of callus consolidation persisting beyond 9 months without evidence of healing progression is classified as nonunion [7].

**FIGURE 1:**
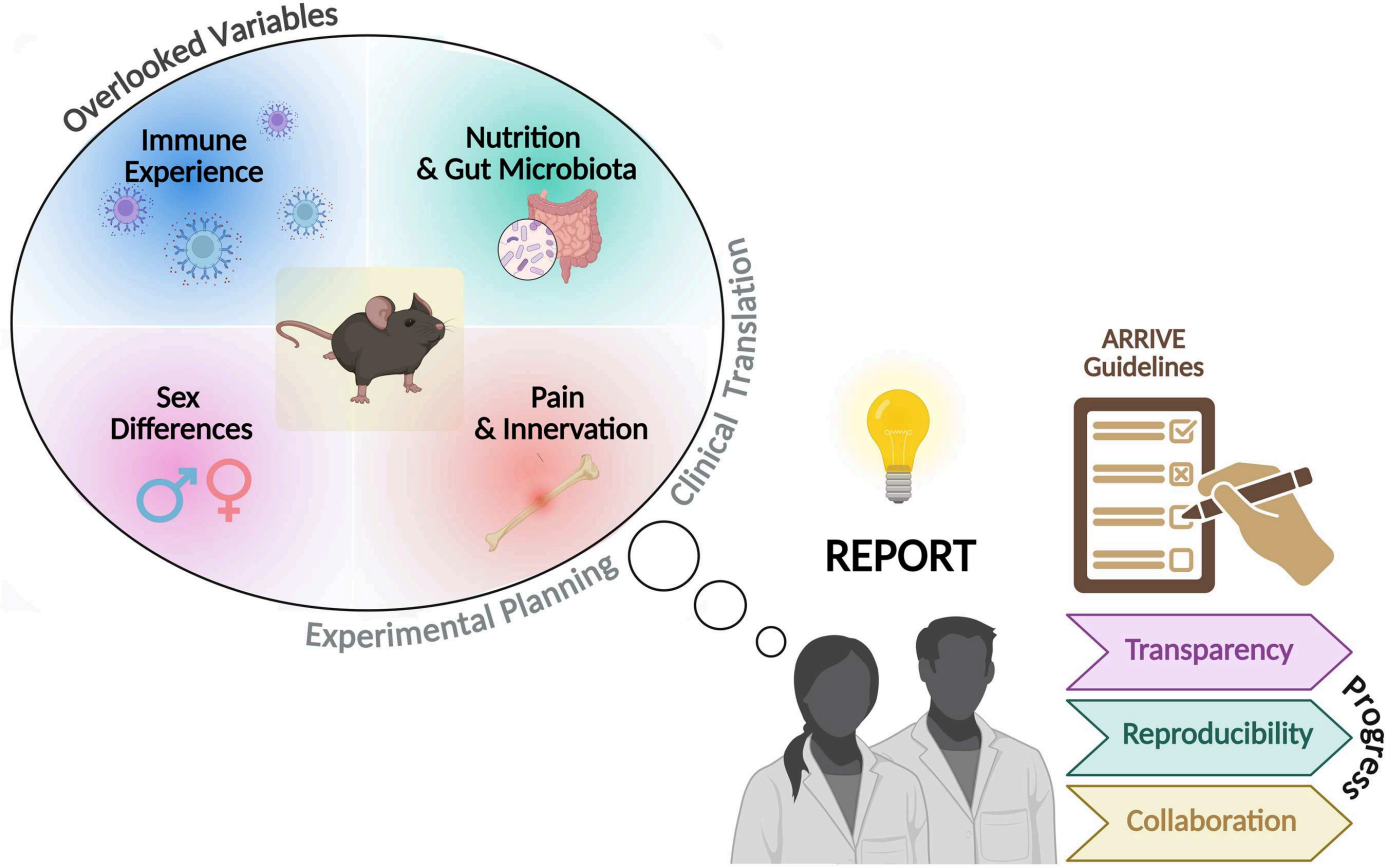
Improving clinical translation in preclinical fracture healing research. Schematic overview of key biological and environmental variables that influence fracture healing outcomes, namely immunological experience, sex differences, nutrition and gut microbiota, and pain and innervation. Integrating these factors into experimental planning and reporting them rigorously according to ARRIVE guidelines will strengthen reproducibility, collaboration, and clinical translation. Created in BioRender. Capobianco, C. (2026). https://BioRender.com/gijnb3y.

Biologic variation and environmental factors can greatly impact the fracture healing process; however, these variables are largely disregarded in preclinical models, contributing to a lack of translation from bench to bedside. Here, we aim to highlight key considerations for improving preclinical animal models to better replicate the demographics, biologic variation, and environmental factors that shape healing outcomes across diverse patient populations (Figure 1).

## 2 Considering Immunological Experience in Fracture Healing

### 2.1 T Cells in Fracture Repair

The stages of fracture repair are heavily governed by immune cells, with neutrophils and macrophages predominant at early timepoints, while lymphocytes arrive in a two‐wave fashion—first during the initial inflammatory phase and again during bony callus formation to regulate osteoclastogenesis and osteoblast function [6, 8]. Disruption of this immune regulation can lead to non‐unions or delayed healing [9]. While preclinical models have recapitulated key aspects of the immune infiltration observed in patients, animals are typically housed in specific pathogen‐free environments, rendering their immune systems naïve compared to those of patient populations. These naïve immune systems lack the repertoire of memory cells that develops upon pathogen exposure, instead exhibiting neonate‐like immune systems [10]. This divergence in immunological experience, particularly within adaptive immune populations such as T cells, can cause alterations in healing that are not captured by use of immune‐naïve preclinical models.

Regulatory T cells (Tregs; CD4+/Foxp3+) have been shown to promote fracture healing by supporting mesenchymal stromal cell proliferation and osteogenesis, and their depletion has been demonstrated to compromise bone formation [11]. In contrast, terminally differentiated CD8+ effector memory T cells (Temra) are detrimental to healing [12, 13]. Unlike Tregs, Temra cells are generated through repeated pathogen exposure and can infiltrate injured tissues independent of specific antigen recognition, leading to their overactivation and accumulation at the fracture site, where they heighten inflammation to a chronic and damaging degree [14]. Notably, Schlundt et al. demonstrated that the ratio of Tregs to Temra cells is a key determinant of healing outcomes [13].

The composition of a patient's T cell repertoire reflects their unique lifetime immunological experience. As individuals age and accumulate pathogen exposures, their pool of naïve lymphocytes diminishes and memory cell populations expand, driving a pro‐inflammatory phenotype associated with poor healing outcomes in elderly patients [15].

### 2.2 Preclinical Models to Recapitulate Patient Immune Experience

While it is increasingly recognized that adaptive immunity impacts tissue repair and bone homeostasis, this is poorly reflected in preclinical models where animals are often kept in Specific Pathogen‐Free (SPF) conditions and are therefore unable to develop true immune experience. These mice instead exhibit neonate‐like adaptive immune systems characterized by a repertoire of predominantly naïve lymphocytes [10]. To address this gap and better recapitulate the immune experience of patient populations, several models have been proposed. Some studies have utilized a humanized peripheral blood mononuclear cell (PBMC) mouse model of bone healing in which mice receive naïve and experienced PBMCs from human donors to recreate the immune profile of patients [15]. In this model, donor blood is characterized as “naïve” or “experienced” based on Temra percentage, with greater than 36% considered experienced. Mice then receive tail‐vein injections of donor blood prior to osteotomy. While this approach provides a means to assess the effect of immune experience on healing, it relies on donor cells being recruited to the fracture site and can be both costly and time‐consuming.

Alternative approaches have sought to allow mice to acquire immune experience more naturally through active pathogen exposure. Methods have included repeated infection, cohousing with pet store mice, exposure to soiled bedding, and transitioning mice from SPF to conventional housing conditions [16, 17].

**FIGURE 2:**
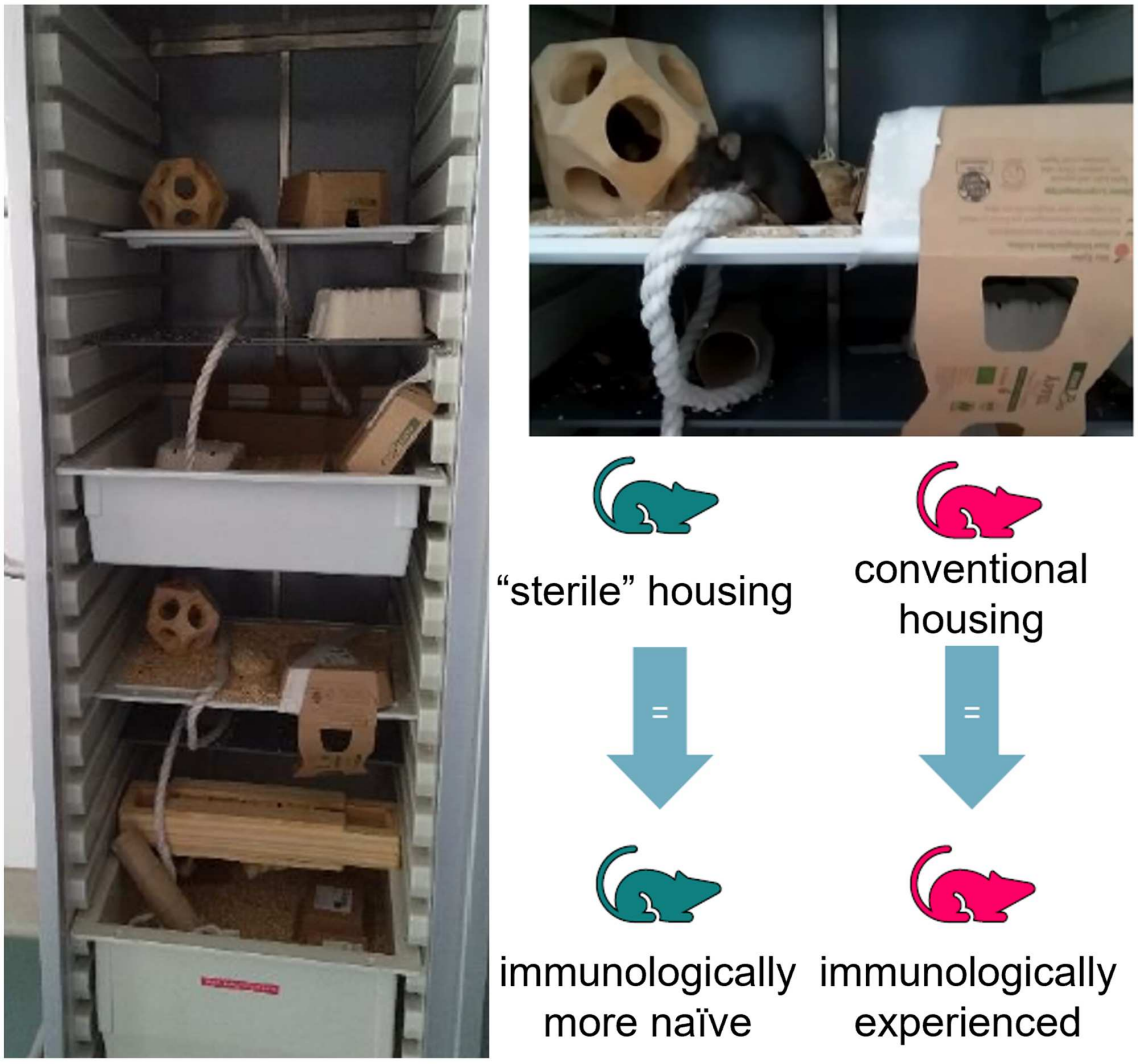
Modeling immunological experience through conventional housing conditions. Representative images of a conventional housing/social enrichment setup used to increase environmental microbial exposure and promote the development of immunological experience in mice. Compared with mice maintained under “sterile” or specific pathogen‐free housing conditions, conventionally housed mice are exposed to a broader range of environmental and social microbial stimuli, resulting in a less naïve and more immunologically experienced immune profile. This approach provides a preclinical strategy to better approximate the immune experience of human patient populations.

The team around Dr. Schmidt‐Bleek at the Berlin Institute of Health at Charité University Hospital Berlin has developed a model that diverges from the current SPF standards for mouse care. In this model, mice are housed in cages without filtered lids, allowing airflow throughout the facility, and are handled without PPE, enabling natural pathogen exposure. Additionally, a social enrichment component has been incorporated in which female mice from various cages are allowed to interact within a shared cabinet for a set period of time, effectively mimicking the natural ecosystem of human social interactions and pathogen transmission (Figure 2). Over the course of 2 years, mice housed under these conditions successfully developed a robust Temra cell population with a diminished naïve T cell compartment compared to their SPF‐housed counterparts. Importantly, these immunologically experienced mice exhibited delayed bone healing, as evidenced by decreased bone volume and mechanical properties alongside increased pro‐inflammatory cytokines, further recapitulating the healing phenotype observed in aged patients [18].

### 2.3 Implications on Patient Treatment

To illustrate the implications of immune experience on therapeutic outcomes, naïve mice received a transfer of CD4+ Tregs prior to osteotomy in an effort to shift the Treg/Temra ratio. In these mice, Treg transfer appeared to improve bone volume fraction, suggesting enhanced bone healing. However, when immunologically experienced mice received the same CD4+ Treg transfer, the effects were mixed: animals with a low CD8 effector cell‐to‐Treg ratio showed significantly enhanced bone healing, whereas mice with a high CD8 effector cell‐to‐Treg ratio showed worse healing outcomes than untreated controls. In these mice, the addition of Tregs appeared to have a detrimental effect due to an overwhelming pro‐inflammatory environment, which would have altered their phenotype from beneficial to detrimental. This study is key in demonstrating the importance of recapitulating immune experience in preclinical models—findings from naïve mice alone would not only fail to predict therapeutic outcomes in humans, but also suggest treatments that are potentially detrimental [13].

## 3 The Sex and Gender Gap in Fracture Research

Biological sex and gender are distinct but interrelated variables. Because this section focuses largely on preclinical animal models, we primarily use the term sex when referring to experimental variables, while recognizing that both sex‐ and gender‐related factors influence fracture risk, healing, and treatment outcomes in human populations.

### 3.1 Reporting of Sex‐Specific Differences

While progress has been made to include women in clinical trials—most notably through the NIH Revitalization Act of 1993, which mandated the inclusion of women and minorities—only 5%–14% of clinical studies examine outcomes by individual sex [19]. Women remain severely underrepresented in research due to exclusion criteria, including risk of pregnancy, hormonal fluctuations, and biased study design. Waltz et al. further identified that exclusion from phase I clinical trials reflects discriminatory themes, including mistrust of women to prevent pregnancies, risk to the fetus, and the financial burden of potential pregnancies [20]. This systematic underrepresentation has led to severe disparities between the number of women recruited to trials and those who bear the burden of disease. For example, while women account for 60% of individuals with psychiatric disorders, they represent only 42% of clinical trial participants [21]. Further disparities arise in clinical diagnosis, as illustrated by the “Mr. Fit” study, in which no women were recruited, resulting in a poor clinical understanding of cardiovascular disease presentation in women and contributing to prevalent misdiagnosis of heart attacks in women more than 50 years later [22].

While men have a predominant fracture risk at younger ages (below 50) [1], assessment of lifetime fracture risk demonstrates overall increased fracture incidence in women over the age of 50 [23]. Despite this, clinical trials remain male‐dominated, with only 34% of all orthopedic studies, both clinical and basic science, reporting sex‐specific outcomes [24]. Further assessment reveals that only 62% of studies report the sex of the animals or human donors in cartilage research, of which 56% report male‐only data [25]. In bone research this is even more pronounced, with 80% of studies reporting no sex or male‐only data [26]. Wolter et al. further revealed that up to 24% of fracture mouse studies specifically failed to report animal sex [27]. This limited sex reporting, lack of transparency, and no standardized all‐sex inclusion across studies have contributed to conflicting conclusions, with some studies reporting increased non‐union risk in men, others in women, and still others finding no sex effect or reporting accelerated healing in males [28, 29, 30, 31, 32]. While these discrepancies might reflect differences in fracture types, phenotypes, age, and experimental or clinical conditions, routine sex reporting and inclusion of both sexes would enable more meaningful cross‐study comparisons.

### 3.2 Influence of Sex on Fracture Healing

The influence of sex on fracture repair is often confounded by factors such as differences in body weight, limb size, age at time of injury, fixator type, and animal model. Two studies performed on 12‐month‐old Sprague–Dawley rats illustrate this complexity. In the first study using males and females of significantly different weights, no difference in healing was observed with a rigid fixator; however, faster healing and the strongest sex effect were observed in males when a semi‐rigid fixator was used. This illustrates how mechanical stability can influence healing outcomes and raises the possibility that weight differences between sexes may alter mechanical loading, thereby confounding the observed sex effect [33]. In the second study, where males and females were weight‐matched at the outset and stabilized with an external fixator, females demonstrated compromised callus mechanical competence and reduced callus cross‐sectional area relative to bone throughout follow‐up [34]. Of consideration, 12‐month‐old rats were used because males and females are comparable in weight at this age, highlighting potential limitations in achieving similar weights at earlier ages. Together, these studies suggest that when weight is controlled for, a true biological sex effect on fracture healing persists, while also highlighting that mechanical stability and body weight are important confounding factors that should be carefully considered and reported.

Broader analysis across mouse and rat fracture models reveals that the majority of studies examining sex generally report accelerated healing in males compared to females, though the magnitude of these effects varies. A closed, un‐stabilized mouse tibia fracture model indicated only modest influences of sex prior to fracture and during remodeling [35], whereas studies using pin‐stabilized femur fractures [36, 37] and femur osteotomy models [38] reported more pronounced healing in males, evidenced by increased total and bone volume, mechanical stiffness, and changes in osteogenic pathways. These differences additionally reflect model‐specific effects as femur and tibia fracture may respond differently to sex‐related factors. Notably, body weight is not consistently reported, and males and females are seldom weight‐matched, limiting the ability to fully interrogate this variable. However, in studies where animals have been weight‐matched (which is challenging in rodents), sex differences remain evident, suggesting that the observed effects extend beyond mechanical loading alone [34, 36]. Furthermore, analysis of female hormonal fluctuations and menopausal effects has demonstrated that estrogen substantially impacts bone quality and healing through its modulation of immune cell activity, inflammatory pathways, and the regenerative potential of female skeletal stem cells [37, 39, 40].

Despite ARRIVE guidelines requiring researchers to report animal sex, this is frequently overlooked, with many studies failing to report sex or focusing exclusively on males. The studies discussed here highlight distinct differences in healing between males and females and underscore the critical need for routine sex reporting in preclinical fracture healing research. They further reveal the importance of considering fracture model and mechanical stability as potentially confounding variables and urge researchers to consistently collect and report animal weight. Where feasible, studies should include both male and female cohorts to dissect sex‐specific variance in injury presentation, healing, and therapeutic response, ultimately improving the translation of patient‐targeted treatments to the clinic.

## 4 Fracture‐Related Pain and Neuroimmune Modulation

Acute pain serves as an important indicator of bone fracture, alerting the body to injury, discouraging use of the affected limb, and guiding recovery. However, persistent pain lasting longer than 3 months is considered indicative of poor healing or non‐union [41]. Certain fractures are particularly prone to chronic pain in patients, with 90% of vertebral body fragility fractures and up to 78% of lower extremity fractures resulting in chronic post‐fracture pain [41, 42, 43]. While NSAIDs reduce inflammation and can alleviate pain, they have been shown to be detrimental to fracture healing [44], and opioids have therefore become the standard of care for post‐operative fracture pain management [45, 46]. Inadequate prescribing guidelines and frequent opioid use have contributed to increased substance abuse and pediatric opioid‐related hospitalizations [45, 46]. Here, we discuss advances in understanding pain, its purpose and underlying mechanisms, and emerging methods to study it, with the goal of developing more targeted therapeutic approaches to reduce fracture‐associated pain without reliance on systemic analgesics.

### 4.1 Innervation and Fracture Healing

Bone is a densely innervated tissue, with sympathetic and primary sensory fibers distributed throughout the periosteum, cortical bone, and marrow cavity. Sympathetic fibers are predominantly located in the bone marrow cavity, while sensory fibers are more abundant in the periosteum [41]. These sensory nerves extend from the dorsal root ganglia and guide skeletal progenitor expansion during development, with increased nerve growth factor (NGF)‐TrkA signaling occurring during periosteal activation and mechanical loading [47, 48]. Mechanical loading of uninjured mouse limbs has been shown to increase NGF expression by osteoblasts and promote subsequent bone formation via Wnt/β‐Catenin signaling in vivo, while inhibition of TrkA decreased Wnt/β‐Catenin signaling and osteogenesis under axial loading conditions [47]. Post‐fracture, NGF is highly upregulated in periosteal progenitors, accompanied by increased TrkA+CGRP+ nerve sprouting [48]. The importance of innervation during fracture repair is further underscored by findings that loss of innervation delays callus revascularization and ossification post‐injury [48, 49]. Xu et al. additionally demonstrated that bone‐innervating DRG neurons undergo dynamic changes post‐injury [49]. These findings have translational implications, as local NGF injections post‐fracture have been shown to increase callus vascularization and accelerate mineralization [50, 51]. Overall, these studies demonstrate a clear role for innervation in fracture repair and highlight potential therapeutic avenues for accelerating healing. However, despite the established necessity of innervation, its contribution to chronic pain and approaches for its treatment remain incompletely understood.

### 4.2 Identifying New Pain Testing Methods and Repair Outcomes

Standard pain testing methods, such as hind paw reflexive avoidance assays (von Frey and Hargreaves), are suboptimal for assessing pain in fracture models, as they measure involuntary reflexes to mechanical or thermal stimuli rather than true pain perception [52]. These assays are additionally prone to variability and bias introduced by animal handling and, in some cases, manual scoring.

Machine learning‐based approaches have become increasingly popular for gait and kinematic assessment. Traditional gait analyses, such as inked footprint methods, provide information on stride length and placement but constrain natural movement by directing animals along specific paths and are cumbersome to quantify manually, introducing variability and inconsistency [41]. A newer technology for gait and behavior phenotyping, the BlackBox Bio, addresses these limitations by utilizing machine learning for automated tracking of free movement, providing over 100 output parameters derived from sensors, cameras, and mirrors to comprehensively assess animal movement [53].

Given the large number of output parameters, recent studies have focused on identifying the behavioral metrics most relevant to fracture healing outcomes. Using the BlackBox, Layne et al. performed longitudinal behavioral testing on mice that underwent either pin‐stabilized or un‐stabilized tibia fractures, tracking behavioral differences between fracture groups and sexes from 5 to 35 days post‐fracture compared to uninjured controls [54]. Principal component analysis was applied to identify parameters that distinguished groups, with striking differences observed in behaviors such as paw pressure, toe spreading, weight‐bearing potential, and step speed. However, to address the complexity of the multidimensional behavioral datasets, the authors further developed a graph theory–based unified metric that integrates all gait and kinematic parameters into a single measure of fracture healing, reconciling variables that often change asynchronously during recovery and providing a more holistic assessment of functional restoration [54]. Notably, sex‐specific differences revealed that females walk at a faster baseline rate than males, highlighting how sex effects on gait could be misinterpreted as accelerated healing unless data are normalized to individual baselines.

Biomarkers have also been interrogated as minimally invasive and cost‐effective indicators of fracture healing status, with potential for clinical translation to enable early detection and intervention of fractures at risk for delayed or non‐union. Working et al. established serum Collagen X as a biomarker of bone volume, finding it to be associated with functional recovery in vivo [35]. Ongoing efforts are focused on identifying neural biomarkers of post‐fracture pain, utilizing, for example, in vivo brain imaging with two‐photon fluorescence microscopy to quantify the activation of pain‐processing centers [55]. Future integration of histological analyses, biomarker assessment, and functional outcomes will provide a more comprehensive framework for identifying biological patterns associated with increased pain and impaired healing.

### 4.3 Translating Preclinical Functional Recovery Outcomes to Patient Care

This preclinical framework for gait and weight‐bearing analysis is highly clinically relevant because successful fracture healing depends on restoration of mobility and mechanical limb loading [41, 56, 57]. Unlike soft tissue pain, skeletal pain is directly exacerbated by movement and loading of the injured limb, impairing locomotion and rehabilitation [56]. Clinical biomotion studies demonstrate that gait asymmetry, weight‐bearing, and kinetic loading parameters are reliable indicators of fracture healing and return to function [58, 59, 60, 61]. Importantly, these deficits can persist long after radiographic union, suggesting that functional recovery captures healing impairments not identified through imaging alone [61]. Furthermore, both biomotion studies and radiographic healing outcomes currently lack a direct connection to fracture‐related pain.

Patient‐reported pain is a strong clinical indicator of poor healing, yet validated patient‐reported outcome measures are not routinely collected in clinical practice. This creates a critical gap in understanding how pain and functional outcomes correlate with radiographic healing. As medicine moves toward precision care, there is an opportunity to integrate multimodal kinematic data collection, patient‐reported pain outcomes and radiographic scoring to guide post‐fracture management and identify early deviations from normal healing to enable timely intervention. Although fracture injuries are regularly imaged throughout the course of healing, the established mRUST scoring method for grading long bone healing is not routinely applied in the clinical setting [62]. This represents an opportunity to retroactively analyze existing imaging data and generate scores that can be correlated with patient‐reported pain and functional outcomes, provided that clinicians consistently collect such measures. Advancing patient care and improving pain management while reducing reliance on opioids will ultimately require broad cross‐disciplinary collaboration among anesthesiologists, orthopedic surgeons, and researchers.

## 5 Insights Into Nutrition and Gut Microbiota

The human body harbors numerous commensal microbiomes that contribute to normal physiological function. While the anatomical location of a microbiome—whether in the stomach, colon, oral cavity, skin, or hair—largely determines its composition, the microbiota is also highly individualized. Evidence suggests that the microbiota may be inherited from the mother and is further shaped by extrinsic factors, including diet, environment, age, pharmaceuticals, and bodily injury. Despite considerable inter‐individual variation, the gut microbiome remains relatively stable throughout an individual's lifetime in the absence of major lifestyle changes, such as moving abroad [63].

The microbiota plays an important role in maintaining human health, and alterations in its composition can lead to poor health outcomes. Disruption of the gut microbiome, termed dysbiosis, can drive increased inflammation and disease. For example, the presence of *Fusobacterium* in the colon has been shown to promote pro‐inflammatory signaling and is associated with increased colorectal cancer risk [64]. Gut microbiome dysregulation has additionally been linked to obesity, liver disease, and rheumatoid arthritis [64]. Despite growing evidence implicating the gut microbiome in bone homeostasis and fracture healing, its role is seldom assessed in clinical practice or preclinical models [65].

### 5.1 Bidirectional Interactions of Fracture and Gut Microbiota

Preclinical studies provide evidence that the gut microbiota influences fracture repair. Several preclinical model systems have been developed to investigate this relationship, including germ‐free mice devoid of all microorganisms. However, this approach can be cost‐prohibitive, technically challenging to perform fracture surgeries in sterile isolators, and causes developmental defects (i.e., underdeveloped immune system). A more practical and inexpensive alternative is the use of broad‐spectrum antibiotics prior to fracture to achieve near‐complete microbiome depletion. This approach, however, carries risks, including selection of antibiotic‐resistant bacteria, fungal overgrowth, and off‐target effects of antibiotics on host physiology [66]. Studies utilizing the antibiotic‐mediated depletion model have demonstrated delayed fracture healing with reduced bone volume fraction, tissue mineral density, and weight‐bearing potential, underscoring the importance of the gut microbiota and its composition in facilitating bone repair [67, 68].

Bone fractures are traumatic events that can significantly alter gut microbiota within as little as 3 days post‐fracture, alongside notable changes in body weight. Both young (3‐month‐old) and middle‐aged (15‐month‐old) mice experience post‐fracture weight loss; however, while young mice rebound within 1 week, aged mice lose approximately 15% of their body weight by 3 days post‐fracture and fail to recover even after 2 weeks. While both age groups exhibit microbiota shifts following fracture, aged mice experience more dramatic changes, characterized by a pro‐inflammatory shift with increased *Lactobacillus* and decreased *Bifidobacterium* abundance [69]. Additionally, treatment of aged mice with *Bifidobacterium longum* was found to improve fracture healing, increase bone volume fraction, and enhance mechanical properties [70].

As the gut microbiome actively modulates fracture repair, recent studies have begun targeting it through dietary interventions. Treatment with specific probiotics, including *Akkermansia muciniphila* and *Lactobacillus gasseri*, as well as probiotic blends has been shown to improve fracture parameters, including bone volume fraction, tissue mineral density, and mechanical properties [71, 72]. Together, these findings demonstrate the translational potential of dietary modifications, such as probiotic supplementation, to modulate the gut microbiota and improve fracture healing outcomes.

### 5.2 Microbiota Considerations in Preclinical Models

Preclinical studies are frequently conducted in mice housed in vivariums across different institutions worldwide, where variations in diet and environmental exposures inevitably alter the gut microbiota. Given the known implications of the gut microbiome for fracture healing, unaccounted microbiota differences likely contribute to the poor reproducibility of in vivo experiments across laboratories. For example, the gut microbe segmented filamentous bacteria (SFB) has been shown to induce Th17 cell accumulation, potentially altering systemic immune responses [73, 74]. Dar et al. demonstrated that SFB+ mice exhibit increased γδ T cell activation and enhanced intestinal Th17 trafficking to the fracture callus, resulting in improved fracture healing, bone formation, and mechanical properties, including stiffness and ultimate torque [67]. Critically, a landmark study in 2009 established that C57BL/6 mice from Taconic Farms are SFB+, whereas C57BL/6 mice from Jackson Laboratories are SFB− [75], demonstrating that the mouse strain alone is insufficient to standardize experimental conditions and that the vendor‐specific microbiome composition can substantially influence experimental outcomes.

To mitigate variability attributable to differences in the gut microbiome, the following practices are recommended. Vivarium diet should be carefully considered; while most facilities use chow diets composed of agricultural ingredients (e.g., ground wheat, ground corn, and dehulled soybean meal), these diets can exhibit considerable batch‐to‐batch variation in nutrients, heavy metals, and phytochemicals, all of which can impact the composition of the gut microbiota [76, 77]. In contrast, purified diets with precisely defined and consistent ingredient quantities are preferable for reproducibility across institutions. Fecal samples should be collected and sequenced to characterize the microbiome, with collection time kept consistent within a study and reported due to circadian variations in microbiome composition [78]. Housing density should also be considered as many preclinical rodent models are coprophagic, which can facilitate microbiota transfer among cage mates and contribute to the so‐called cage effect. Although microbiome studies often use single housing to limit this, a more appropriate approach may be to decrease housing density (e.g., two mice per cage) to minimize cage‐level microbiome effects [79] while avoiding the stress associated with social isolation [80]. Adherence to these guidelines will support greater reproducibility across preclinical fracture healing studies.

## 6 Conclusion

The studies presented here collectively highlight the profound impact of biological variation and environmental factors on fracture healing outcomes and underscore the persistent gap between preclinical models and the complexity of human patient populations. Current preclinical models have been invaluable for mechanistic studies of fracture repair, particularly because they allow careful control of biological and environmental variables. As the field continues to advance translation, it may be useful to consider how commonly used experimental conditions, including age, sex, SPF housing, immune experience, diet, and standardized environments, relate to the broader diversity of patients that these models are intended to represent.

Key findings across the sections presented here demonstrate that immunological experience, specifically the balance between regulatory and effector memory T cell populations, critically shapes fracture repair outcomes and therapeutic responses. Sex‐specific differences in healing are evident but frequently obscured by poor reporting practices and confounding variables such as body weight and mechanical stability. Pain and innervation play active and underappreciated roles in the repair process, and emerging behavioral assessment tools offer promising avenues to bridge functional and tissue‐level outcomes. Finally, the gut microbiota emerges as a powerful and modifiable contributor to fracture healing, with dietary interventions showing translational potential. Together, these findings call for a shift toward more physiologically relevant preclinical models and greater standardization in reporting practices.

## 7 Take‐Home Message—REPORT, REPORT, REPORT

The persistent gap between preclinical research and clinical practice may reflect not only scientific and methodological challenges, but also challenges in communication and translation. As researchers, we have a responsibility not to operate in isolation, but to foster collaborative efforts, promote reproducibility through rigorous reporting, and design studies that consider how our models reflect the diversity of patient populations. The ARRIVE guidelines represent the current standard for reporting preclinical animal studies [81], and we strongly urge researchers to adopt and rigorously apply them. In addition, the findings presented here highlight several variables that are frequently overlooked yet critically influence fracture healing outcomes. Beyond the technical details already captured by ARRIVE, such as fracture model, fixation method, surgical standardization, and outcome measurements, we wish to emphasize the following specific reporting requirements for preclinical rodent fracture healing studies (Table 1). While preclinical rodent models are essential for understanding musculoskeletal biology, we acknowledge that there may be limitations and difficulties in controlling variables in the rodent models. Thus, researchers must carefully consider the best animal model according to the context of their preclinical studies and the objectives and outcomes of their experiments.

**TABLE 1:** Overlooked biological and environmental variables recommended for reporting in preclinical rodent fracture healing studies.

Animal demographics	Housing and environmental conditions	Immune and microbiological status
Sex of all animals. Age at time of surgery. Body weight at time of surgery. Whether male and female cohorts are weight‐matched. Animal movement/behavior.	Housing condition (SPF vs. conventional). Vendor and animal supplier. Number of animals per cage. Diet type (agricultural vs. purified, with composition reported where possible).	Immune status where relevant (naïve vs. immunologically experienced). Antibiotic treatment prior to or during the study. Pain management protocol and pain assessment methods. Hygiene status of the facility and/or fecal microbiome sequencing data (recommended as a future standard).

## Author Contributions

Design of topic and structure: Annemarie Lang, Melanie Haffner‐Luntzer, Matthew J. Silva, and Christina Capobianco. Writing manuscript text: Christina Capobianco and Annemarie Lang. Revising manuscript text: Katharina Schmidt‐Bleek, Melanie Haffner‐Luntzer, Chelsea Bahney, Joseph L. Roberts, and Matthew J. Silva. Approval of final manuscript: Annemarie Lang, Melanie Haffner‐Luntzer, Matthew J. Silva, Christina Capobianco, Katharina Schmidt‐Bleek, Chelsea Bahney, and Joseph L. Roberts.

## Data Availability

Data sharing is not applicable to this article, as no new data were created or analyzed in this study.
